# High-throughput quantification of the effect of DMSO on the viability of lung and breast cancer cells using an easy-to-use spectrophotometric trypan blue-based assay

**DOI:** 10.1007/s00418-019-01775-7

**Published:** 2019-02-16

**Authors:** Sarah Musa Hammoudeh, Arabella Musa Hammoudeh, Rifat Hamoudi

**Affiliations:** 1 Sharjah Institute for Medical Research, College of Medicine, University of Sharjah, 27272 Sharjah, UAE; 2 Department of Clinical Sciences, College of Medicine, University of Sharjah, Sharjah, UAE

**Keywords:** Cell count, Trypan blue, Spectrophotometric assay, DMSO, High-throughput screening

## Abstract

One of the main aspects investigated in potential therapeutic compounds is their effect on cells viability and proliferative ability. Although various methods have been developed to investigate these aspects, these methods present with shortcomings in terms of either cost, availability, accuracy, precision, or throughput. This study describes a simple, economic, reproducible, and high-throughput assay to quantify cell death and proliferation. In this assay, adherent cells are fixed, stained with trypan blue, and measured for trypan blue internalization using a spectrophotometric absorbance plate reader. Corresponding cell counts to the absorbance measurements are extrapolated from a standard curve. This assay was used to measure the effect of dimethyl sulfoxide (DMSO) on the viability of breast and lung cancer cells. Decrease in cell count associated with the increase in DMSO percentage and exposure time. The assay’s results closely correlated with the conventional trypan blue exclusion assay (Pearson correlation coefficient (*r*) > 0.99; *p* < 0.0001), but with higher precision. The assay developed in this study can be used for various applications such as optimization, cell treatment investigations, proliferation, and cytotoxicity studies.

## Introduction

Cell counting is a common, important practice in cell culture used on daily basis for different applications. The increased incorporation of cell count in various techniques and assays imposed growing needs for higher levels of cell-count accuracy, precision, and throughput than those offered by the originally developed and standardized methods. Hence, various alternative approaches were developed aiming at fulfilling these requirements and overcoming original methods’ shortcomings. These approaches were based on various concepts including automation, flow cytometry, impedance, tetrazolium salts, methylene blue, microscopy, fluorescence, and protein quantification.

Originally, the use of haemocytometers and counting chambers was the standardized cell-counting approach. However, due to their various limitations (i.e., low accuracy, low precision, low throughput, high probability of variations, and susceptibility to various human error sources), the use of these methods became limited to simple applications (Ongena et al. 2010; Tucker et al. 1994). An improved, automated modification of these methods was developed aiming at increasing the cell-count throughput. However, it failed to overcome the other shortcomings due to the similarities in sample preparation and mounting onto the special counting slides/chambers (Camacho-Fernandez et al. 2018). Furthermore, despite the various adjustment options, automated counters remain prone to fail in realizing varying cellular shapes and sizes pooled in the same sample as well as out-of-focus cells in different focal planes (Camacho-Fernandez et al. 2018).

Other approaches aiming at increasing the accuracy and precision of counting were based on passing and counting each cell in the sample discretely (e.g., flow cytometry and impedance-based Coulter cell counting) (Collins et al. 2010; Coulter 1953; DeBlois and; Bean 1970). However, the use of these approaches requires special devices and reagents that are relatively costly and often unavailable for use. Furthermore, some of these methods focus on measuring cell number and size, but lack the ability to distinguish viable cells and cell aggregates.

Less demanding approaches with high accuracy and precision include the use of colorimetric assays (e.g., tetrazolium salt-based approaches) (Mosmann 1983; Roehm et al. 1991). However, these methods require the use of reagents and kits that are costly. Furthermore, as those approaches are based on the measurement of the colored formazan products, they are prone to artifacts affecting the NAD(P)H-dependent cellular oxidoreductase enzymes’ activity and formazan production (Maioli et al. 2009). These assays could as well be cell-type dependent with varying efficiencies. Colorimetric methylene blue-based counting assays were developed as simple cell-counting assays with relatively high throughput and relatively low cost (Felice et al. 2009; Oliver et al. 1989). However, the requirement of the elution step in these processes results in variations in readings and requires intensive optimization efforts (Felice et al. 2009).

In accordance with these shortcomings, we aimed to develop an easy method that would only require daily used reagents in cell culture while still providing a close efficiency to that of the other developed approaches. Applications of such an approach can vary to include proliferation studies, cytotoxicity studies, seeding count optimizations, and treatment optimizations. The capacity of this method as a cell counting and toxicity assay will be assessed through the application of DMSO to lung and breast cancer cells. DMSO is a common reagent used in various cell-culture applications including the preparation of treatments and cryopreservation. However, increased exposure to DMSO can lead to increased cell damage resulting in pseudo-effects in treatments as well as cell loss in cryopreservation.

## Materials and methods

### Cell culture and standard curve preparation for the assay

A549 and MDA-MB-231 cell lines (ATCC, USA) were cultured in RPMI-1640 media supplemented with 10% FBS and 1% penicillin–streptomycin (Sigma, USA) and maintained at 37 °C and 5% CO2. Cell suspension concentrations were prepared in 8 serial dilutions at a ratio of 3:4 starting with 350,000 cells/ml (Table 1). Cells were then seeded in 2 sets of triplicates (one set for traditional counting and the other for the trypan blue colorimetric assay) in a 96 well plate, 100 µl per well. Additional arbitrary concentrations (Table 2) were chosen to assess the efficiency of the assay in estimating cell count and were seeded with or without 5% dimethyl sulfoxide (DMSO; Sigma, USA), similarly in triplicates. The 96-well plate was left for 10–15 min in the biological cabinet before transferring to the incubator to avoid aggregation of cells in the middle of the well. A549 cells were incubated for 6 h, whereas MDA-MB-231 cells were incubated for 20 h. Cells were monitored throughout for adherence and normal morphology adaptation and the experiment was stopped (fixation/trypsinization) once these conditions were fulfilled.

**Table 1:** Cell concentrations seeded to constitute the standard curve

Concentration number	Standard curve serial concentrations (cells/ml)
1	350,000
2	262,500
3	196,875
4	147,656
5	110,742
6	83,056
7	62,292
8	46,719

Following the first concentration, a ratio of 3:4 is used to construct the subsequent serial dilutions

**Table 2:** Chosen arbitrary values seeded to assess the performance of the assay

Concentration number	Chosen arbitrary values (seeded count (cells/ml) ± DMSO treatment)
1	200,000 – DMSO
2	200,000 + DMSO
3	150,000 – DMSO
4	150,000 + DMSO
5	100,000 − DMSO
6	100,000 + DMSO
7	50,000 − DMSO
8	50,000 + DMSO

Each concentration is seeded with (+) or without (−) DMSO treatment to investigate the potential use of the assay for cytotoxicity studies

### Traditional trypan blue cell counting

Cells were harvested with 0.05% Trypsin–EDTA (Sigma, USA) and enzymatic activity was neutralized with FBS-containing RPMI-1640 Media. Cell densities were measured using a haemocytometer after the addition of trypan blue. Densities were then adjusted for the final count per well by multiplying by the suspension volume in each well.

### Trypan blue staining

The cells were fixed with 4% Paraformaldehyde (PFA) (Sigma, USA) (prepared in PBS) for 20 min at room temperature followed by 2 washed with PBS. The cells were then stained with 0.1, 0.25, or 0.4% Trypan Blue (Sigma, USA) diluted in PBS for 10, 30, or 60 min at room temperature. The cells were then washed twice with PBS to completely remove any remaining trypan blue residues and avoid an artifact signal. The absorbance was then measured using BioTek’s plate reader ELx808 at absorbance of 450 nm, 490 nm, and 630 nm.

### Microscopy

Some of the images were acquired with Olympus IX53 inverted microscope equipped with Olympus DP75 camera (resolution of 5760 × 3600 pixels and pixel size of 5.86 × 5.86 µm) with Cell Sens Entry software (version 1.17). Images were acquired with 10X (numerical aperture 0.50) objective lenses at 1600 × 1200 pixels and 72.0 1/in resolution in both X and Y axis. The rest of the images were acquired using Olympus IX73 inverted microscope with Olympus DP22 camera (resolution of 1920 × 1440 pixels and pixel size of 3.69 × 3.69 µm) and DP camera acquisition software. Images were acquired with 4X (numerical aperture 0.13) or at 1920 × 1440 pixels resolution.

### Statistical analysis

*T* test and one-way ANOVA tests were conducted to statistically analyze the cell-count data and identify differences between the results of the different counting methods. The significance was taken to be *p* < 0.05. Excel was used to conduct the goodness-of-fit test (*R*^2^) as a part of the standard curve linear regression analysis. Otherwise, all statistical analyses (including Pearson’s correlation) were carried out using GraphPad Prism.

## Results

### Optimization of the trypan blue assay parameters

The use of this assay requires the optimization of multiple parameters: (1) standard curve range and ratio of serial dilutions; (2) standard curve plating duration; and (3) duration of fixing and staining.

To optimize the standard curve range, multiple concentrations of cells approaching the two extremes (full confluency and very low confluency) were plated in a 96-well plate. Accordingly, the rough borderlines of the standard curve range were determined, ~ 35,000 cells/well at full confluency and ~ 5000 cells/well at low confluency, for both cell lines. This range was then used to identify the dynamic range of the assay by testing its accuracy at the two previously identified borderlines. Subsequently, a serial dilution ratio (3:4 dilution starting from 35,000 cells/well) was chosen to create a standard curve spanning the chosen range (Table 1). The cells were plated according to the dilutions and monitored regularly for signs of adherence and adaptation of natural morphology in culture. Once the cells fulfilled these conditions (after 6 h for A549 cells and 20 h for MDA cells), the cells are directly fixed for staining to avoid increase in cell count.

**Fig. 1:**
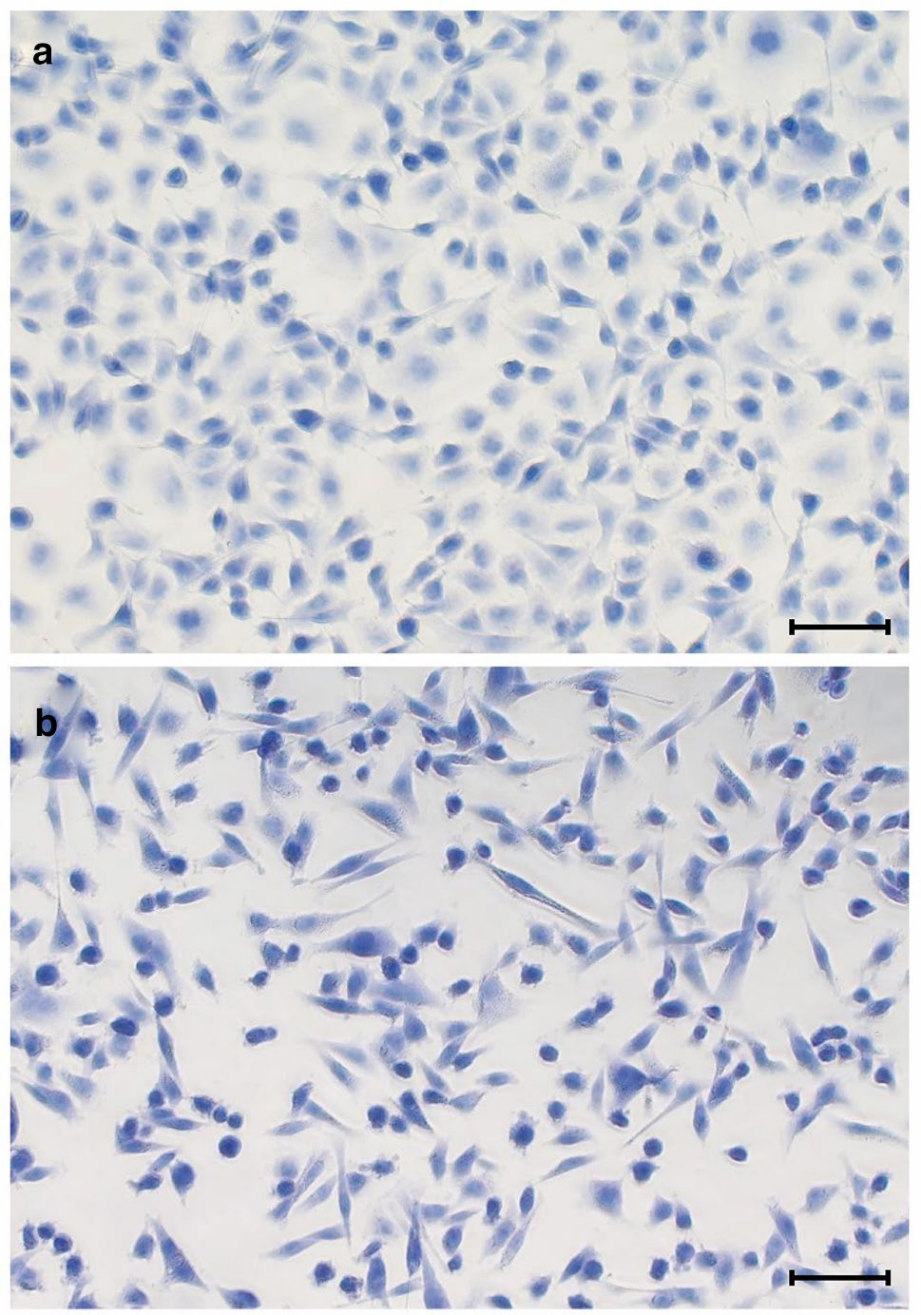
Dye absorption by **a** A549 and **b** MDA-MB-231 cells after fixation and staining with trypan blue. Scale bars representing 100 µm. Images acquired with 10X magnification through Olympus IX53 inverted microscope

Beyond fixing the cells, the application of PFA sufficiently permeabilizes the cell membrane to allow for the entrapment of trypan blue intracellularly and the full coverage of the cell body (Fig. 1). This method of staining results in dye density signals proportional to the volume and thickness of the cell; rounded cells are less spread but thicker resulting in a darker shade of blue and the opposite for cells that adapted to the normal morphology. This balance between color density and surface area coverage compensates for differences in cell morphologies and allows for a better cell-count estimation.

**Fig. 2:**
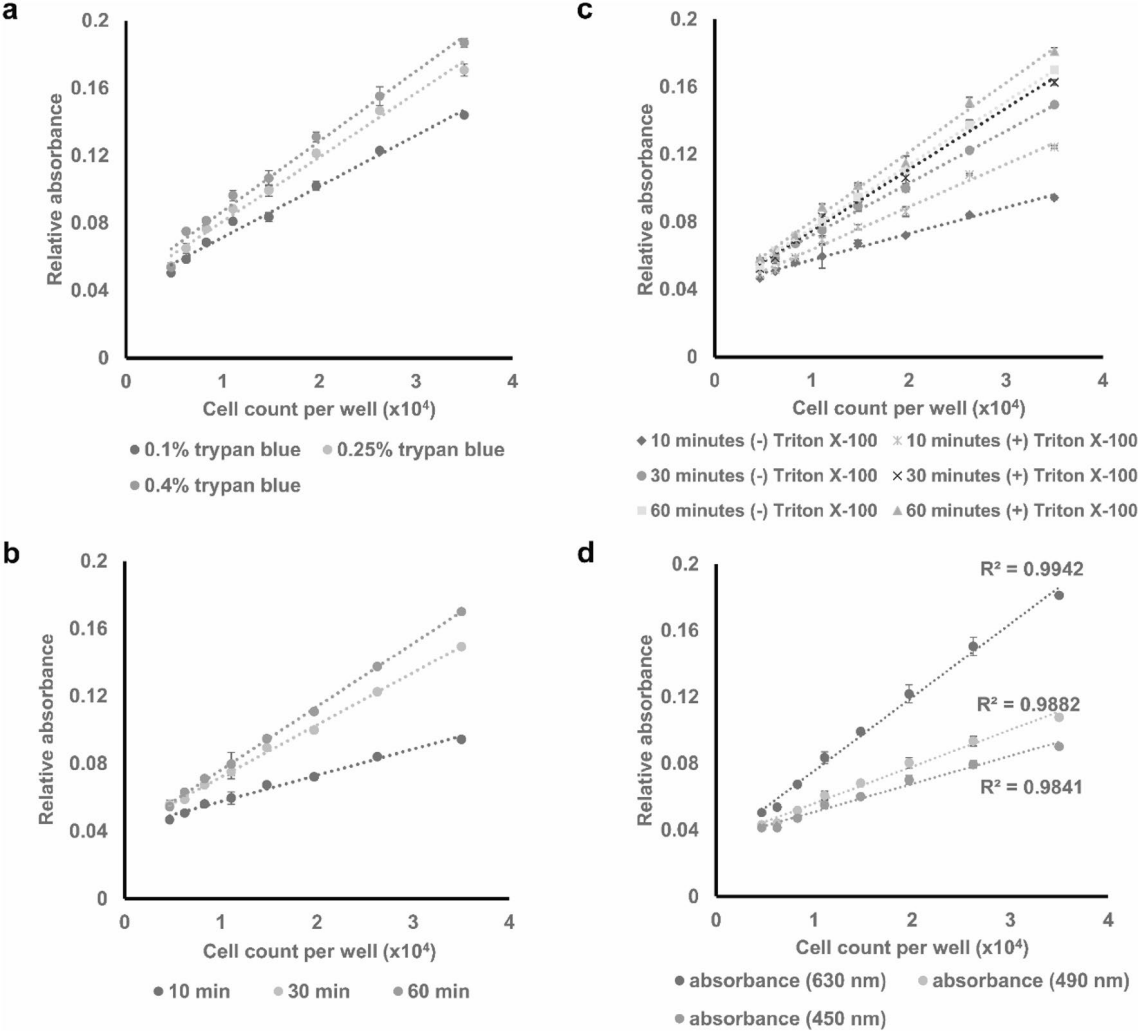
Optimization of trypan blue staining parameters including **a** trypan blue concentration (0.1, 0.25, and 0.4% trypan blue), **b** staining duration (10, 30, and 60 min), **c** pre-permeabilization with 0.5% Triton X-100, and **d** absorbance acquisition wavelength. Data points presented as mean± SEM

Staining duration was optimized over 0.5, 1, 2, 5 h, and overnight. As shown in Fig. 2, although the trypan blue signal could be visualized even at shorter staining duration, the strength of the signal directly correlated with the staining duration. Therefore, to achieve the best visualization of the cells, overnight staining was applied to the subsequent experiments. Inefficiencies in the trypan blue internalization into the cells might require optimization of the fixation method and staining duration. Otherwise, one might consider the application of a highly diluted detergent (e.g., Triton X-100) to lightly permeabilize cell membrane and increase dye internalization.

To achieve the optimum staining procedure, several parameters were optimized (Fig. 2). Initially, the staining resulting from ascending concentrations of trypan blue (0.1, 0.25, and 0.4%) was investigated showing a positive concordance between the concentration of trypan blue and dye absorption (Fig. 2a). With the use of the highest concentration, 0.4% of trypan blue, the staining duration was optimized over 10, 30, and 60 min (Fig. 2b). Positive correlation was similarly observed between the staining duration and dye absorption. The additional permeabilization of the cells with 0.5% Triton-X100 prior to the staining was found to further increase trypan blue uptake (Fig. 2c). Therefore, permeabilization with Triton X-100 can be used to reduce the staining duration; 30 min dye uptake by Triton X-100 permeabilized cells closely approaches that of non-permeabilized cells stained for 1 h. Taken together, the optimum staining conditions were found to be staining with 0.4% trypan blue at room temperature for 30 min if preceded by 20 min of Triton X-100 permeabilization or 1 h without permeabilization.

Furthermore, absorbance measurement at the three different wavelengths (450, 490, and 630 nm) was found to provide sufficient signal to estimate cell count, indicating the flexibility of the assay (Fig. 2d). However, as the absorbance measurement at wavelength of 630 nm provided the strongest signal, and the highest correlation and accuracy, it was used in this paper for all subsequent measurements.

### Standard curve accuracy verification

**Fig. 3:**
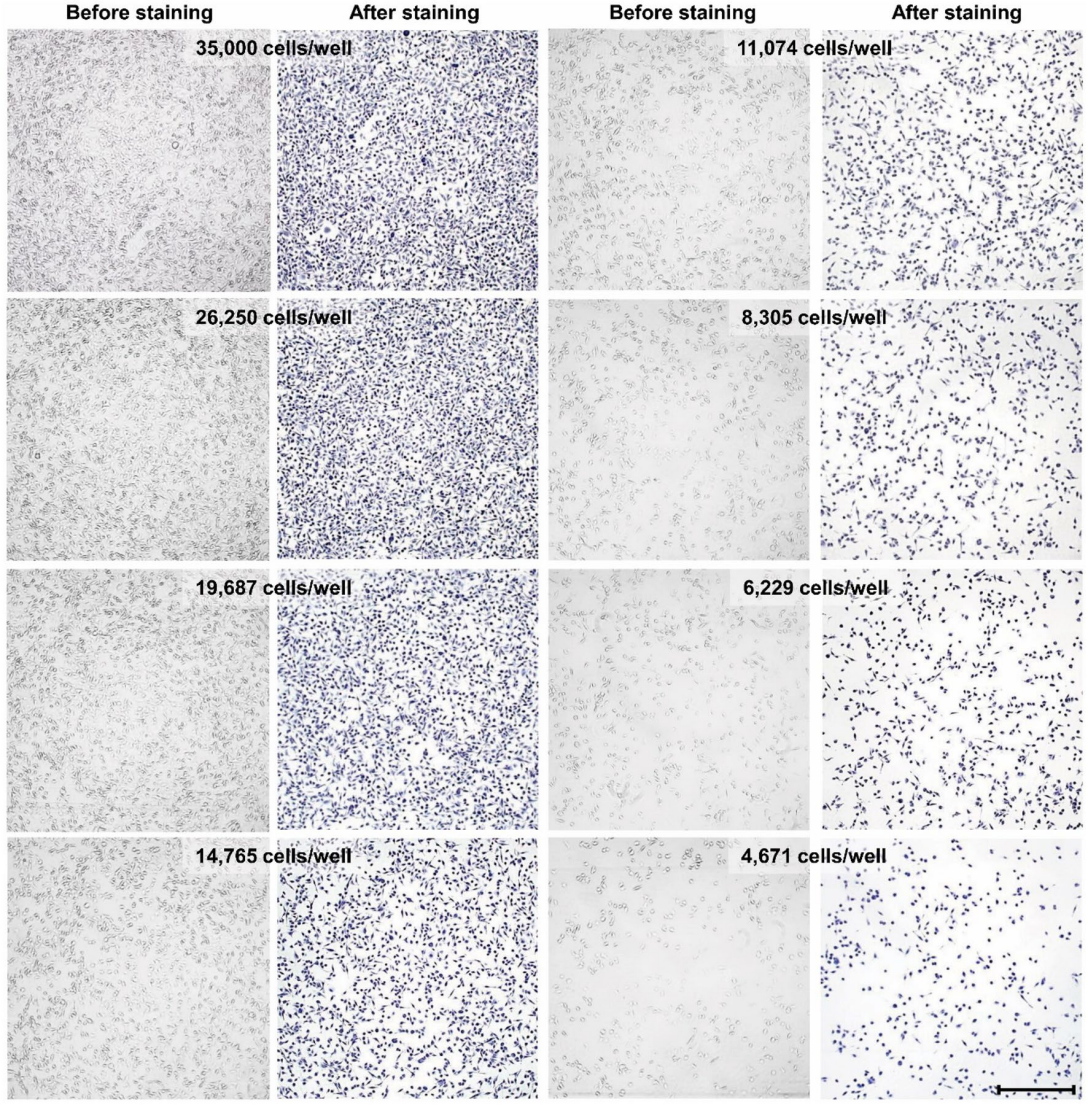
Standard curve serial densities of A549 cells before and after fixation and staining with trypan blue. Scale bars representing 200 µm. Images were taken at a 4X magnification using Olympus IX73 inverted microscope

**Fig. 4:**
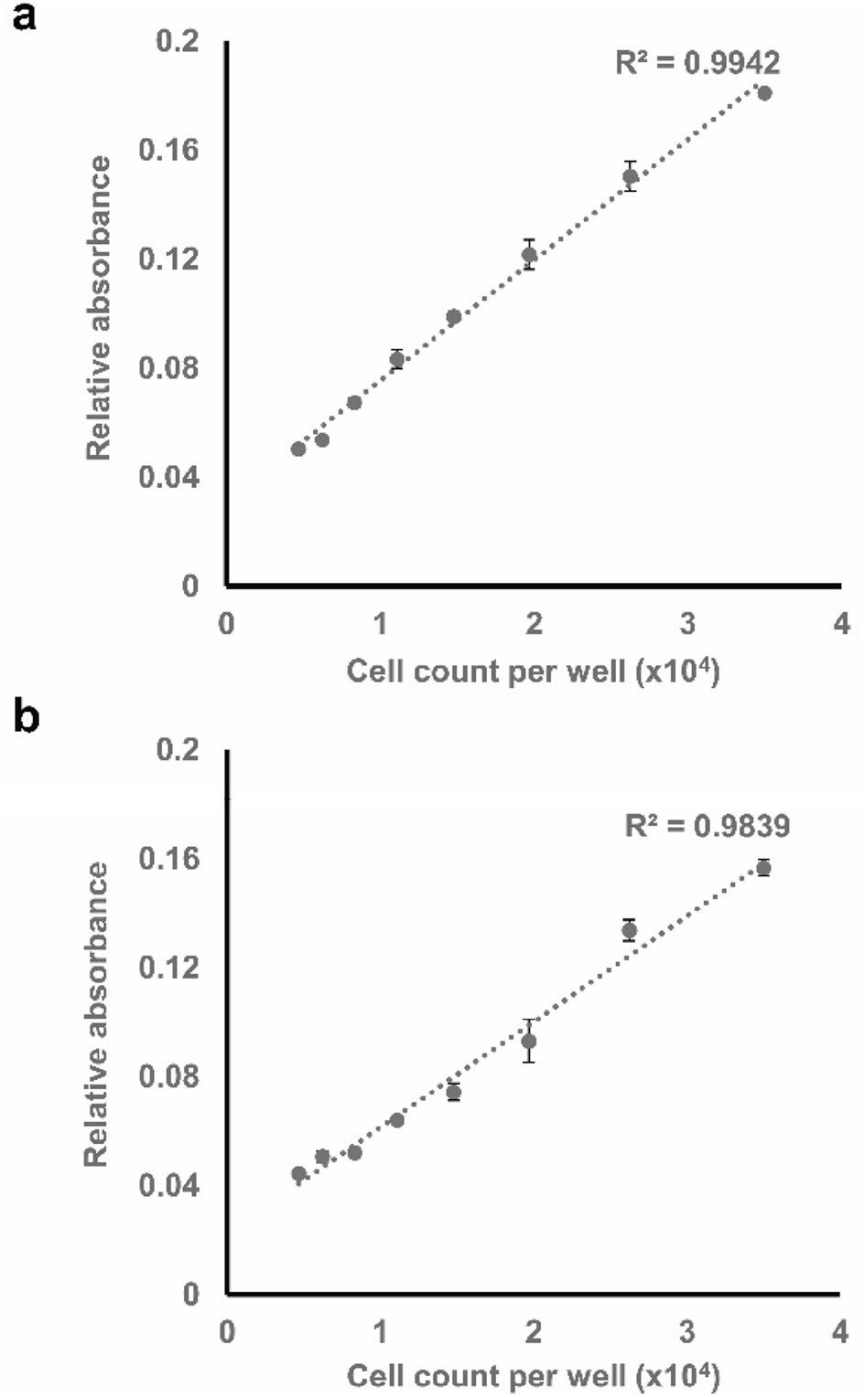
Absorbance standard curve and goodness-of-fit test (*R*^2^) as a measure of correlation for **a** A549 and **b** MDA-MB-231 cells. Data points presented as mean± SEM

The gradual reduction in cell density along the standard curve serial dilutions and the corresponding decrease in trypan blue staining were visually verified using phase contrast microscopy (Fig. 3). The trypan blue absorbance signal was then quantified using BioTek’s plate reader Elx808. As seen in the absorbance measurements of the standard curves for both cell lines (Fig. 4a, b), a significant correlation [correlation results for the A549 cell line: Pearson’s correlation: 0.9971; *p* < 0.0001; goodness of fit (*R*^2^): 0.9942; correlation results for the MDA-MB-231 cell line: Pearson’s correlation: 0.9919, *p* < 0.0001; goodness of fit (*R*^2^): 0.9839] is achieved with low variations amongst the triplicate measures at each point.

### Comparison between cell-count measurements using the conventional haemocytometer and the new trypan blue assay

**Fig. 5:**
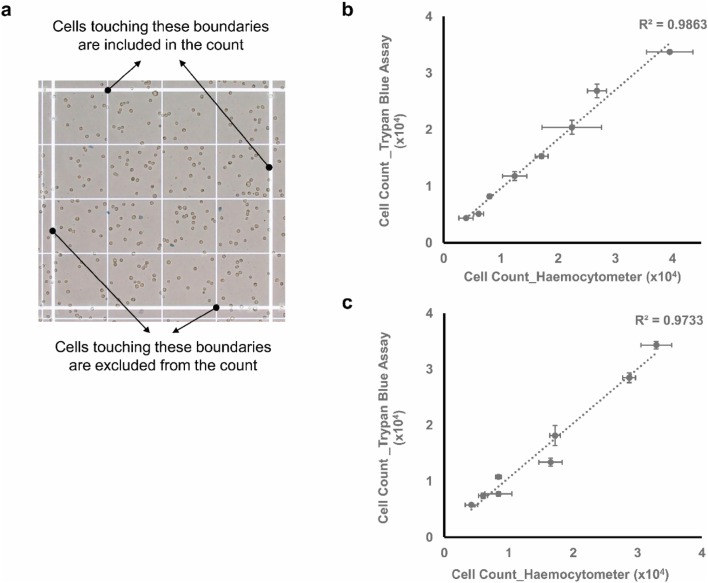
**a** Representative image of hemocytometer grid taken for traditional hemocytometer counting and comparison between cell count, **b** A549 (Pearson correlation: 0.9931 and *p* value for is > 0.0001), and **c** MDA-MB-231 cells (Pearson correlation: 0.9866 and *p* < 0.0001) obtained using haemocytometer and the new trypan blue assay. Data points presented as mean± SEM (horizontal error bars representing SEM of haemocytometer count while the vertical ones representing that of the new trypan blue assay)

Standard curve cell-count measurements were similarly done using the traditional haemocytometer counting method. Several precautions were taken to increase the accuracy and precision of these counts including: (1) ensuring proper detachment of cells and homogenous suspension; (2) conducting triplicate measurements at each point; (3) counting cells in the four outer edges squares of the haemocytometer grid; and (4) including cells only on the top and right edges of each of these squares (Fig. 5a). Furthermore, images were taken of the haemocytometer grid instead of instantaneous counting to avoid cell damage due to prolonged cell incubation at room temperature.

These cell-count measurements were then compared to those of the new trypan blue colorimetric assay. As can be seen in the graphs (Fig. 5b, c), the measurement from both methods was relatively close and fluctuates narrowly around the initially seeded counts. However, in comparison with the haemocytometer’s measurements, the counts from the new assay were found to be more representative of the initially seeded cell counts and more precise in terms of triplicate measurements. This indicates the comparatively higher accuracy and precision of the new trypan blue colorimetric assay.

### Efficiency of the assay in estimating arbitrary samples with unknown counts

**Fig. 6:**
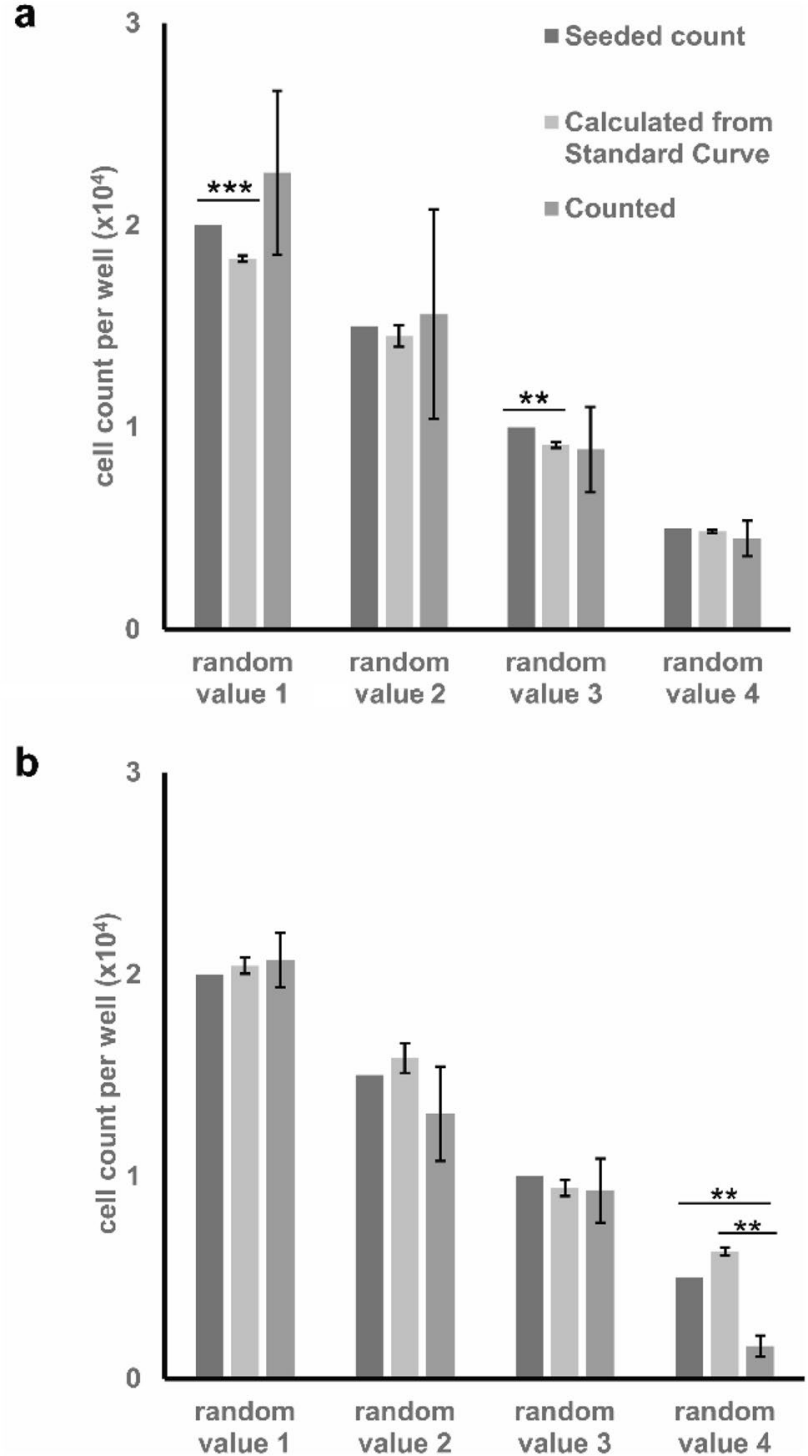
Cell-count measurements of arbitrary seeded counts of **a** A549 and **b** MDA-MB-231 cells using traditional haemocytometer counting (counted) and the new trypan blue assay (calculated from standard curve) in comparison with originally seeded counts. Data points presented as mean± SEM; **represents *p* ≤ 0.01, and ***represents *p* ≤ 0.001

Arbitrary values were chosen to assess the power of the assay and its ability to estimate unknown cell counts. As seen in the graphs of the two cell lines (Fig. 6), the results from the two assays fluctuate closely around the initially seeded counts. However, at multiple data points, the trypan blue assay proved to yield closer cell counts to those initially seeded. Furthermore, the ranges of inter-triplicate variations are significantly smaller in the trypan blue spectrophotometric assay’s measurements.

### Cytotoxicity assay

**Fig. 7:**
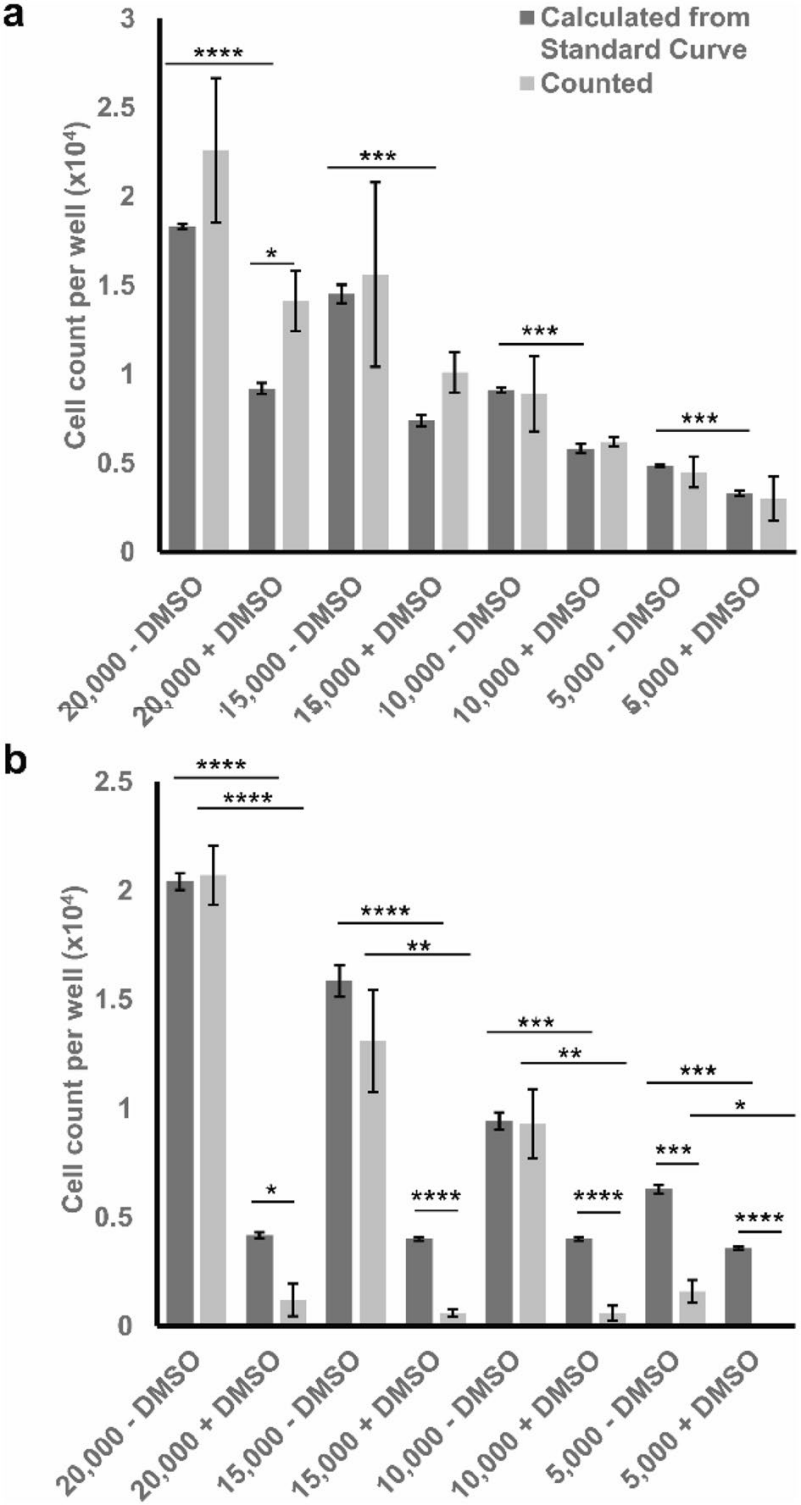
Cell-count measurements of the chosen arbitrary cell counts after treatment with 5% DMSO calculations for **a** A549 cell line and **b** MDA-MB-231 cell line using traditional haemocytometer counting (counted) and the new trypan blue assay (calculated from standard curve). Data points presented as mean± SEM; *represents *p* ≤ 0.05, ** represents *p* ≤ 0.01, *** represents *p* ≤ 0.001, and **** represents *p* ≤ 0.0001

The arbitrarily chosen values above were treated with 5% DMSO to assess the ability of the trypan blue spectrophotometric assay in measuring treatment-induced cytotoxicity. The assay measurements of the DMSO treated cells (Fig. 7) show a clear reduction in cell count, conforming to the results of the standard haemocytometer counting.

**Fig. 8:**
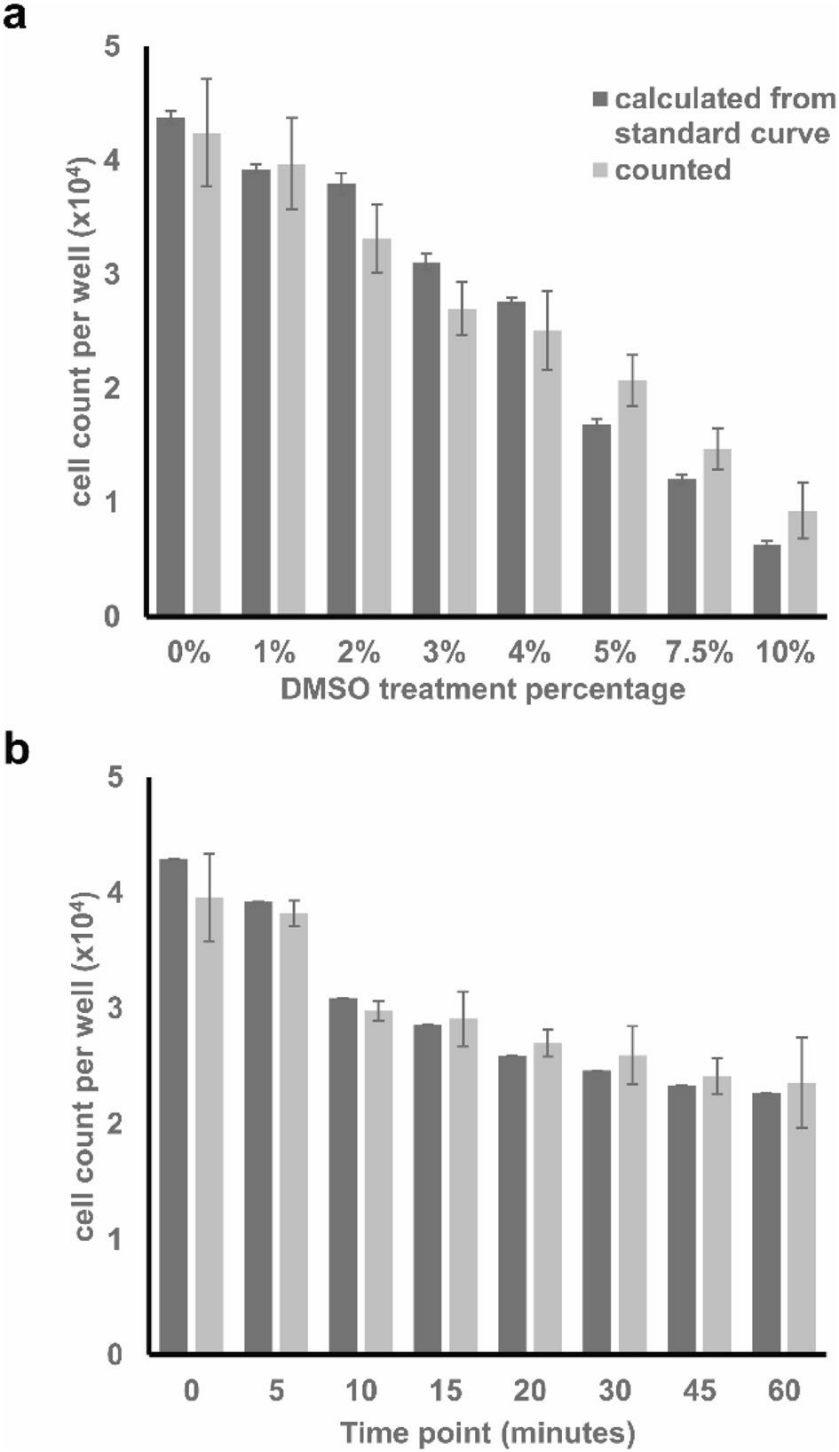
Cell-count measurements of A549 cells treated with **a** 0, 1, 2, 3, 4, 5, 7.5, and 10% of DMSO for 24 h and **b** 10% DMSO for increasing time periods (0–60 min) measured with the traditional hemocytometer counting (counted) and the new trypan blue assay (calculated from standard curve). Data points presented as mean± SEM

Furthermore, the resolution power of the assay was assessed by measuring the cytotoxic effect of 3 consecutive concentrations of DMSO, 1, 2, 3, 4, 5, 7.5, and 10% (Fig. 8a). Starting at an initial count of ~ 40,000 cells per well, a gradual decrease is seen in cell count in correspondence with the increase in the applied DMSO concentration. Hence, this demonstrates the ability of the assay to detect and differentiate between relatively close cell counts. Moreover, the cell-count measurements obtained with the new trypan blue assay positively correlated with those from traditional haemocytometer counting (Pearson’s correlation coefficient: 0.9771; *p* < 0.0001; *R*^2^: 0.9546), verifying the validity of the new assay’s measurements.

### Measurement of the cytotoxic effect of DMSO exposure one range of treatment durations

The assay was used to assess the gravity of damage inflicted on the cells in correlation with the increase in the incubation duration with 10% DMSO—commonly used concentration for cryopreservation. As shown in Fig. 8b, increased incubation with 10% DMSO is correlated with increased damage to the cells. This damage is more drastic in the initial 10 min after which the rate of cell loss decreases. However, the viability of the cells must be taken into consideration as the cells might be damaged regardless of their adherence. These results were found to highly correlate with those of the traditional haemocytometer cell count (Pearson’s correlation coefficient: 0.9947; *p* < 0.0001; *R*^2^: 0.9894), verifying the capacity of the assay to reflect and quantify cell damage.

## Discussion

Although various cell-counting approaches were developed to fulfil application-specific needs, these approaches are either low throughput (e.g., haemocytometer) or relatively cost-demanding (e.g., flow cytometry). In this paper, we present a simple, cost-efficient, time-saving, high throughput, and reasonably precise and accurate assay. The concept behind this assay is to measure cell density by measuring the internalization of trypan blue by fixed, adherent cells. This measurement is then used to infer the cell number in the well by plotting to a standard curve.

As shown in the results’ section, the proposed trypan blue assay provided close estimation of the arbitrarily selected cell counts. Moreover, it efficiently captured the cytotoxic effect of DMSO treatment on cells which positively correlated with the increase in the applied DMSO percentage. The negative effect of DMSO on cell viability was found as well to correlate with the exposure duration. 10% DMSO is a commonly used concentration in preparation of cells for cryopreservation. However, prolonged exposure to this percentage is shown above to induce cell damage proportional to the exposure duration. These results reflect the previous findings on the various mechanisms stimulated by DMSO to induce cell damage in correspondence to its concentration. For instance, DMSO application at concentrations > 10% results in the perforation of the plasma membrane and eventually apoptosis (De Ménorval MA 2010; Notman 2006). On the other hand, lower concentrations of DMSO were found to result in the nuclear translocation of the apoptosis-induced factor (AIF) from the mitochondria, activation of poly-(ADP-ribose)-polymerase (PARP), and stimulation of caspase-3-independent apoptosis pathways (Galvao 2014).

In comparison with the haemocytometer standardized counting, the trypan blue colorimetric assay counting results are relatively higher in accuracy, precision, and resolution. Furthermore, the assay only requires manual counting of a single value (the highest value in the standard curve) which would be used as the reference count for all subsequent calculations. Hence, the assay has a significantly increased throughput and time efficiency. Standard counting with haemocytometer, as well as many of the other approaches, requires the trypsinization and detachment of cells. This process increases the risk of cell loss and affects cell count. In this approach, we eliminated this risk by directly staining and measuring the absorbance on the same cell-culture surface of the 96-well plate.

Moreover, the use of this assay requires reagents used daily in cell-culture practice (i.e., trypan blue and paraformaldehyde) and a simple spectrophotometric plate reader, usually at disposal to most. Therefore, the assay is developed to be user-friendly, economic, and suitable for frequent, simple counting procedures (i.e., optimization experiments, proliferation, and cytotoxicity assessment). To further increase the cost efficiency of the assay, the diluted trypan blue dye can be recycled from the previous experiments. Another advantage of this approach is that in case of weak trypan blue signal, the cells can be simply re-incubated with the trypan blue dye for a longer time period. Furthermore, the experiment plate stained with the trypan blue can be saved for back-referencing by storing it at 4 °C in PBS with the option of re-staining in case dye is eluted.

Due to the cell-volume dependence of the assay, the measurements done through this assay are relatively reproducible. This results in various advantages; for example, the optimized standard curve could be reused for the calculations for subsequent assays on the condition that the fixation and staining processes are identical. This is especially the case with stable cell lines with uniform morphology, adherence, and growth rates. Furthermore, cell populations with irregular shapes would be accounted for in this assay. Shape irregularities will result in changes in cell thickness which will be directly accounted for by changes the trypan blue signal (positive correlation between thickness and signal). Moreover, it might be possible to use the assay for counting cell populations heterogeneous in volume and shape as these differences will be considered in the standard curve and reflect on the count of the target samples; this require on proper homogenous distribution of these cells throughout the suspension. Third, overlapping or clustering of seeded cells would result in a correlative increase in dye intensity. Thus, the overlapped cells would be as well accounted for in the final count of cells per well.

Successfully conducting this assay requires several precautions and requirements: (1) ensuring homogenous distribution of cells on the well surface by allowing the cells to settle and adhere to the plate before transferring it to the incubator; (2) pipetting gently to avoid cell loss due to mechanical pressure; and (3) reducing the incubation time of the cells in suspension before plating to enhance cell adhesion and ensure proper morphology adaptation.

Although this approach has the shortcoming of being a fixed-cell assay, its value lies in its potential use as a proliferation/cytotoxicity assay similarly to, but at a lower cost than currently existing colorimetric assays (e.g., tetrazolium salt-based approaches). The use of this approach can be ideal for optimization procedures requiring multiple repetitions and assessing multiple parameters with wide ranges. In such cases, the use of the relatively expensive approaches would result in a high economic burden. While economic options, such as the haemocytometer, would be impractical due to its low throughput and potential time consumption. Accordingly, this assay can provide measurements relatively better than those of the haemocytometer and at a faster rate and economic cost. Moreover, this assay could potentially be modified and expanded to other cell viability exclusion dyes with similar properties to those of trypan blue according to their availability in the lab, further increasing the feasibility of the assay.
